# Stillbirth with a false-positive lung float test result – an unusual case report

**DOI:** 10.1007/s12024-025-01035-2

**Published:** 2025-07-02

**Authors:** E. Hoffmann, L. Malolepszy, C. Hochscheid, R. Dettmeyer, M. Fritzenwanker

**Affiliations:** 1 Institute of Legal Medicine, Justus-Liebig-University Gießen University Hospital Gießen & Marburg GmbH, Frankfurter Str. 58, D-35392 Gießen, Germany; 2 Institute of Microbiology, Justus-Liebig-University Gießen University Hospital Gießen & Marburg GmbH, Schubertstr. 81, D-35392 Gießen, Germany

**Keywords:** Stillbirth, Lung float sample, Gastrointestinal float sample, *Fusobacterium gonidiaformans*

## Abstract

A male newborn found lifeless raised the question of whether he had lived after birth. The float sample test results of both lungs and the gastrointestinal tract were positive. Microbiological examinations detected the *Fusobacterium gonidiaformans*, an obligate anaerobic gas-forming germ, in the lung tissue and in the heart blood, which caused the float test results to be ‘false-positive’. As far as can be seen, a comparable case has not yet been reported in forensic literature.

## Introduction

In the case of dead newborns, the question of survival outside the womb must regularly be clarified. The lung and gastrointestinal float test was first described by Schreyer in 1682 [1]. Since then, this test has been considered suitable for the detection of peri- and postnatal pulmonary respiration [2, 3]. The cadaveric period is important, as putrefaction can lead to a false-positive lung float test result [4]. Positive results are extremely unusual in stillbirths with a very short post-mortem period, well-chilled cadavers without relevant putrefaction and post-mortem examinations carried out promptly.

## Case report

A 19-year-old pregnant woman with no documented previous illnesses complained of mild abdominal pain on the day before birth and severe abdominal pain on the day of birth. Further symptoms, particularly symptoms of infection such as fever, were not documented. The father of the child had assisted with the home birth and alerted the emergency services. A newborn baby was found dead in a rubbish bag in the bedroom. The parents reported no breathing, umbilical cord pulsation or palpable pulse in the newborn. The on-site forensic examination revealed residual heat in the corpse, partial rigour and visible green rot in the skin of the abdomen. The mother of the child was taken to hospital. She had been found to have very high levels of inflammation.

For better visualisation see Table 1.

**Table 1:**
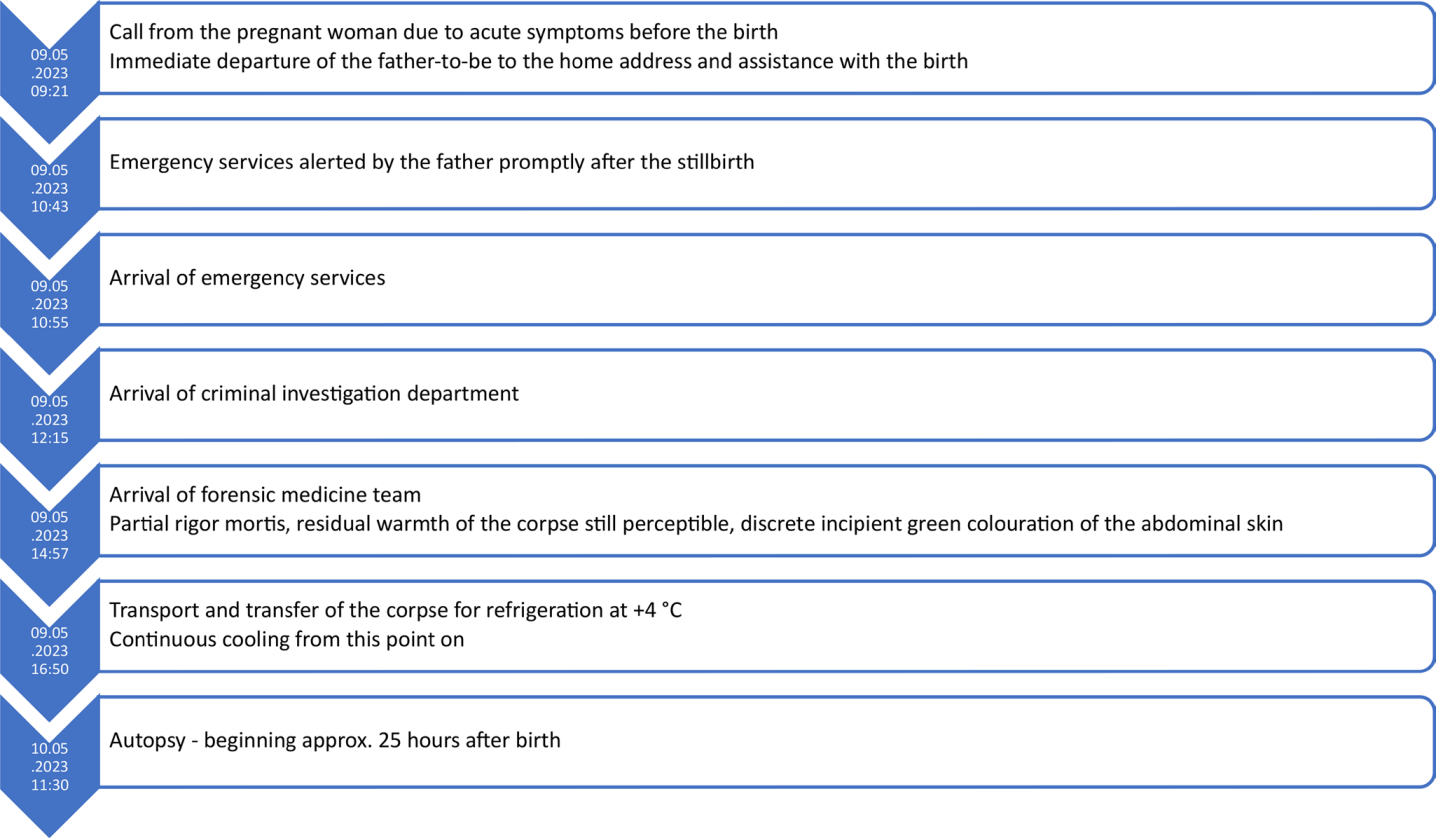
Timeline of the case for better visualisation

### Autopsy

A well-chilled, mature, male newborn (bodyweight 3242 g, crown-heel length 52 cm) in the 8th/9th month of pregnancy was brought for autopsy about 25 h after birth. Signs of maturity: Both testicles descended, hair length 2 cm, abundant vernix caseosa, fingernails at fingertip level. Macroscopically, no signs of gross external violence, especially of fatal significance, no evidence of umbilical cord strangulation. No macroscopic pathological findings in internal organs.

**Fig. 1:**
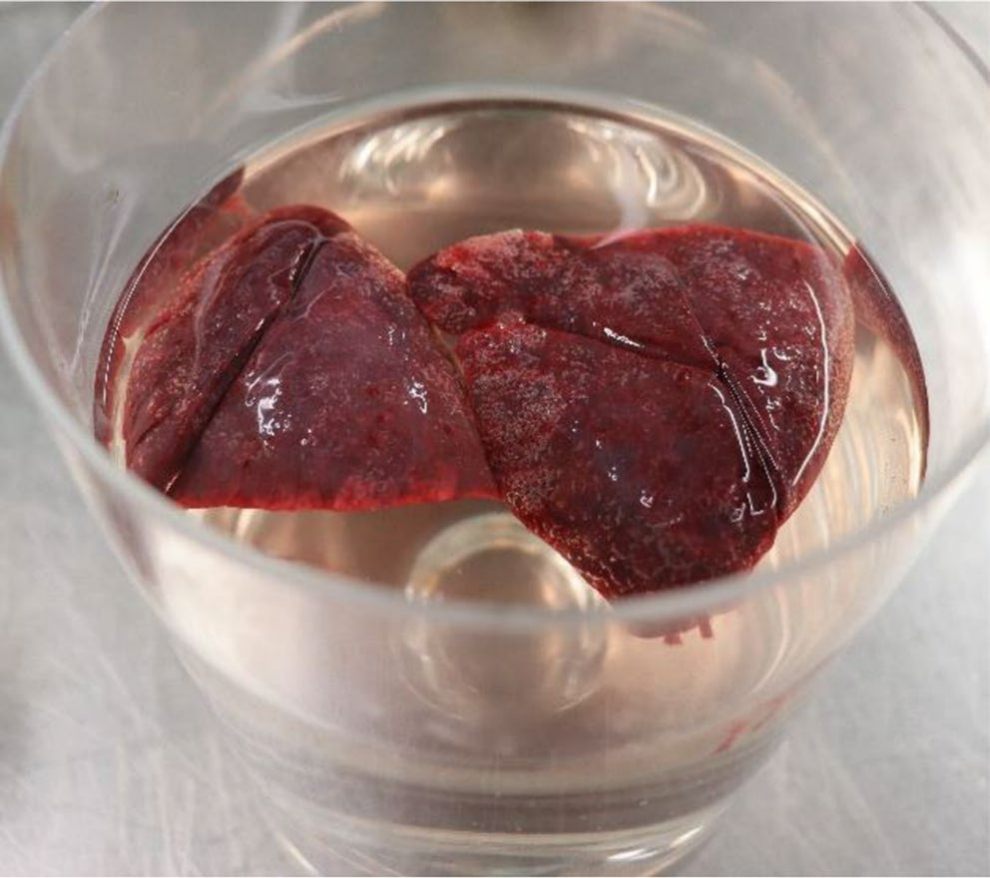
Lungs floating on the water surface with subpleural gas bubbles

**Fig. 2:**
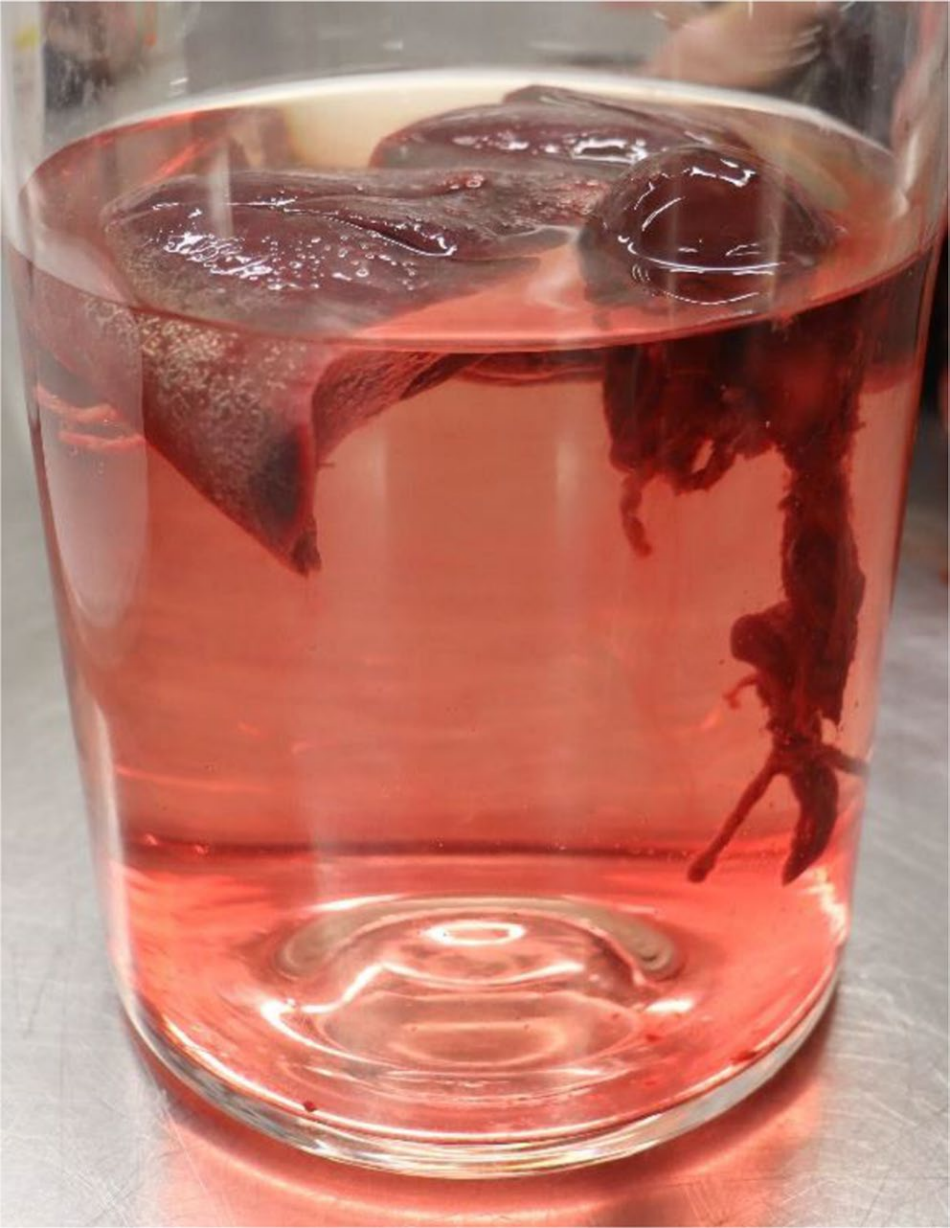
Lungs and gastrointestinal parts floating on the water surface

Slight green mould of the abdominal skin. Subpleural, surprisingly fine-bubble gas inclusions (Fig. 1). A gas crackle was palpable in the lung tissue. The thoracic cavities and abdomen each contained 1–2 ml clear fluid. There was significant gas distension of the small intestine. The float sample test results of both lungs and the lobes of the lungs were positive (Figs. 1 and 2), and the gastrointestinal float sample tested positive (Fig. 2). The placenta (576 g, 16 × 13 × 4 cm) with an eccentrically inserted, three-vessel umbilical cord showed no macroscopical signs of inflammation, no malformations, and no ruptures. A cause of death could not be determined macroscopically.

## Histology

**Fig. 3:**
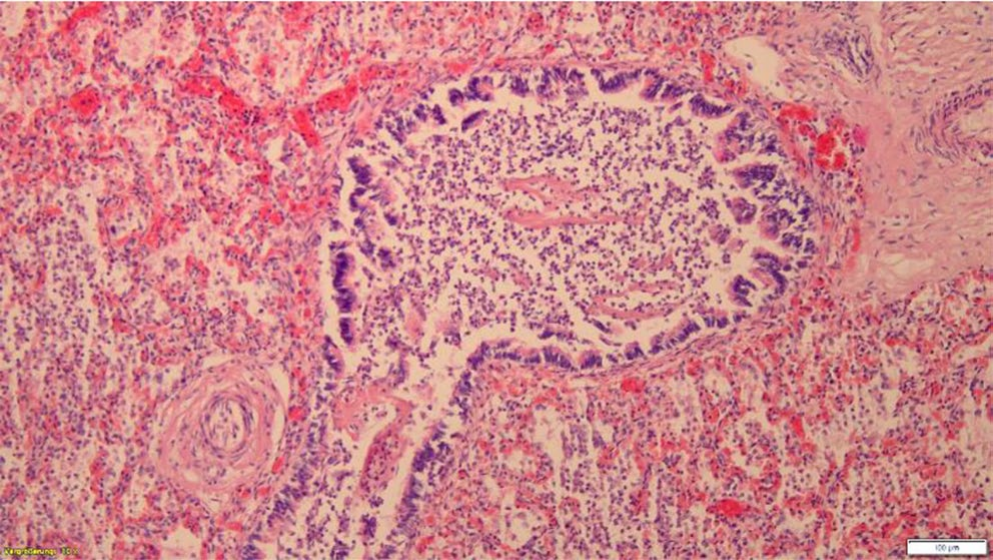
Lung, HE, x100. Bronchus with leucocytes

**Fig. 4:**
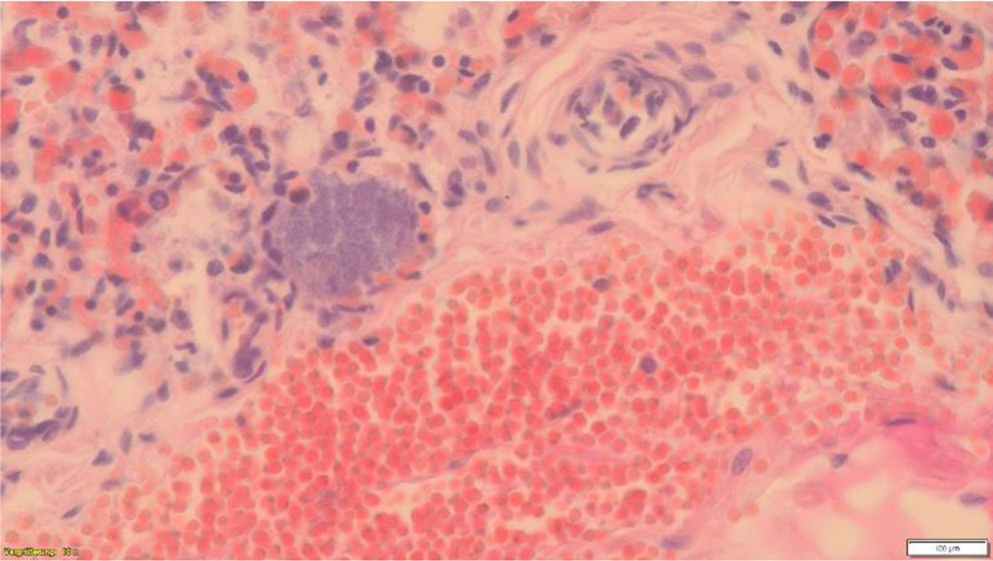
Lung, HE, x400. Bacterial cell nest with leucocytes

**Fig. 5:**
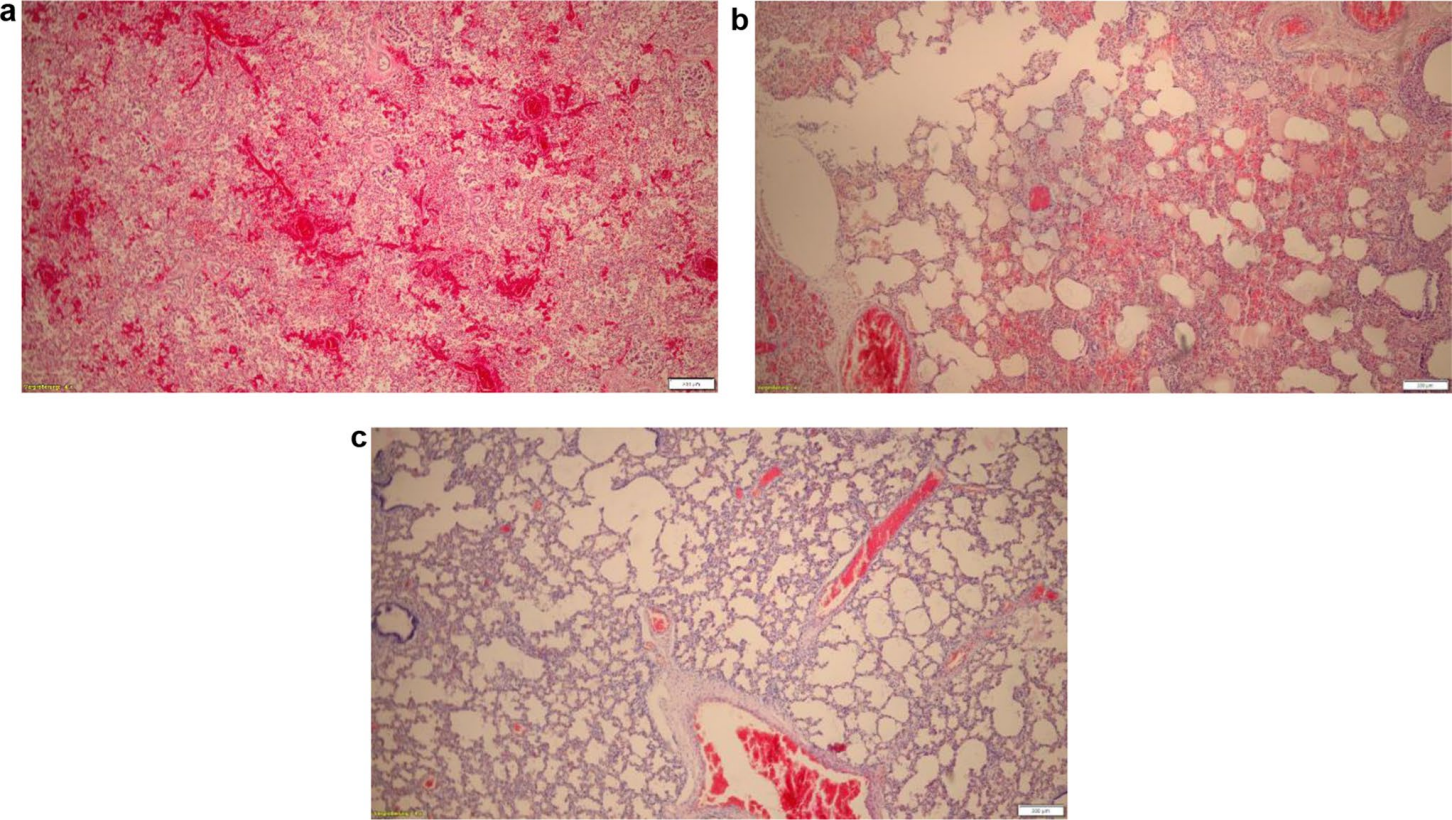
**a**: Lung, HE, x40. Mature stillborn with complex fetal atelectatic lung without ventilated pulmonary alveoli, negative float sample test. **b**: Lung, HE, x40. Mature newborn with incomplete and inhomogeneous, partial gas-infloated alveoli (Current case. Microbiological finding of anaerobic gasformation in the lung tissue: *Fusobacterium gonidiaformans*). **c**: Lung, HE, x40. Mature newborn with almost entirely ventilated lung tissue und positive float sample test.

Lung tissue showed basic physiological anatomy, bacterial cell nests and focal leucocyte accumulations as well as gas-inflated cavities, somewhat emphasised subpleurally and in the periphery of the lung tissue (Figs. 3, 4 and 5a − 5c). In addition, non-ventilated atelectatic lung areas. Physiological amniotic fluid aspiration, not unusual in vaginal delivery, could not be detected. Histologically, no other internal organs showed any pathological findings. No acute bacterial placentitis, omphalovasculitis and/or chorioamnionitis. No placental infarcts. No histological evidence of putrefaction.

### Microbiology

*Fusobacterium gonidiaformans*, gas-forming, obligate anaerobic bacteria [5] were detected in lung tissue and heart blood.

### Toxicology

Chemical toxicology tests and a determination of the blood alcohol concentration were negative.

## Discussion

The autopsy revealed no gross external force, in particular no umbilical cord entanglement. There was no evidence of killing by soft covering or any means of asphyxiation.

On the question of whether the mature [6] foetus died prenatally or postnatally: The so-called lung float test is an established method for determining whether the foetus died outside the womb [2, 3]. In individual cases, this test may not be reliable [7]. An anaerobic germ colonisation can lead to gas formation post-mortem due to putrefaction. This can cause a ‘false-positive’ lung float test result. An appropriate environment and a sufficient time period are required for putrefaction processes and putrefactive gases [8]. In the presented case, the corpse was found quickly, cooled well and autopsied promptly.

The fusobacteria of the gonidiaformans species detected in the lung tissue and heart blood belong to the family of obligate anaerobic, non-spore-forming, gram-negative rods. Fusobacteria are found in the oral and intestinal microbiome. Serious infections in immunocompromised patients are rare, but have been described. *F. necrophorum* and *F. nucleatum* can be detected more frequently, whereas *F. gonidiaformans* is very rarely described but is considered to be related to *F. necrophorum* [5, 9–16]. Typical infections with fusobacteria are chronic infections in the orofacial area (dental, sinusitis, peritonsillar abscesses). Fusobacteria can also occur in secondary infections such as intra-cerebral and intra-abdominal abscesses or thrombophlebitis (Lemierre syndrome, *F. necrophorum*) and, in rarer cases, in pulmonary abscesses. Particularly significant are amniotic infections, both with and without (!) opening of the membranes, which are described with mild symptoms as well as with abortions and septic courses (‘occult’ chorioamnionitis) [17–20]. Transmission of the pathogen from the intestinal microbiome to the foetus is conceivable both haematogenously (in the case of bacteraemia transmitted from the mother) and via infection of the amnion and subsequent oral ingestion by the foetus.

Fusobacteria are considered to be strong producers of hydrogen sulphide [21, 22], which may be relevant in halitosis in purulent dental abscesses. Foetor is also described in some case reports of infections of other organs. The ability to produce H_2_S is implicated as a pathogenicity factor. Mutants lacking this ability are associated with a lower risk of infecting the amnion, placenta, and foetus [23]. In case reports, gas production in various organs has been diagnosed radiologically and described as a possible, yet nonspecific, sign of infections caused by these pathogens [24–30]. In the presented case, such gas production may have caused the false-positive lung float test result. As typical obligatory anaerobic germs, fusobacteria are growing slowly, incubation times of the bacterial family in the laboratory are between 48 hours and 7 days [5], in vitro doubling times between 1 and 3.5 hours have been described [31, 32]. Growth rates in vivo can deviate, as pH, temperature, nutrient supply and local host defence can be significant. When temporal dynamics in Fusobacteria infections are reported, protracted courses are typically mentioned. In Lemierre’s syndrome, for example, a pharyngeal infection is noticed first, and complications due to septicaemia become apparent 1 to 3 weeks later [33].

Due to their fastidious nature, these anaerobic species can be missed in routine microbiological culture. They can also be covered (and subsequently missed by the human eye) by an overgrowth of non-fastidious bacteria, particularly aerobic or facultative anaerobic bacteria like streptococci, staphylococci or enterobacterales, all of which can often be found in autoptic samples. To facilitate growth and detection of anaerobic species we use additional agar plates (Schaedler Anaerobe KV Selective Agar with Lysed Horse Blood, Thermo-Scientific) which we incubate under anaerobic conditions. These plates are supplied with different growth factors that enhance the growth of anaerobic species, and with antibiotics that inhibit the growth of the other aforementioned species.

The fastidious nature of these species and their natural habitat also means that they are not typical laboratory contaminants. As they are part of the human gut microbiota, it is, of course, not completely impossible. However, we found a large number of them in two independent samples (heart blood smear: about one-third of the plate covered by *Fusobacterium* colonies; lung swab smear: complete plate covered with *Fusobacterium* colonies). We consider contamination during the sample cultivation process highly unlikely.

In this case, the obligate anaerobic germs could be detected in the lungs and in the heart blood. As the autopsy was performed promptly and the corpse was well cooled, it can be assumed that the foetus was infected intrauterine before death, possibly days to weeks before birth. A post-mortem infection with the obligate anaerobic pathogens via the respiratory air is practically impossible. Even if one were to assume a postnatal exposure to *F. gonidiaformans*, the macroscopic and histological findings could not be explained, as the anaerobes only grow slowly even under optimal nutrient conditions (the incubation period for bacteriological detection is 48 h or more) [5]. Growth is further slowed down by cooling. Intrauterine oral ingestion via the amniotic fluid or haematogenously is conceivable, resulting in fatal septicaemia shortly before birth.

The presence of gas-forming, obligatory anaerobic germs in the lung parenchyma and the fine subpleural and intrapulmonary gas bubbles, in the absence of faeces, explain a partially atelectatic, prenatally developed lung parenchyma. The largely absent, and to a certain extent physiological, amniotic fluid aspiration also speaks against respiration of the newborn after birth [34]. The intrapulmonary and gastrointestinal gas formation of the germ thus led to ‘false-positive’ lung float tests and a ‘false-positive’ gastrointestinal float test. We considered the term ‘false-positive’ because of the misleading positive findings of the lung- and gastrointestinal float tests, which led to the retrospectively false assumption that the foetus had been alive. The histologically recognisable leukocyte infiltration in the lung tissue extending down to individual clearings of the peripheral bronchi suggests a protracted, gradual infection via the amniotic fluid. To the best of our knowledge, a comparable case — a stillbirth from an intrauterine fatal bacterial infection caused by an obligate anaerobic germ, and resulting in false-positive float samples — has not yet been described in forensic literature.

### Summary

Positive float test results from both lungs and the gastrointestinal tract initially suggested that the mature male neonate was alive. The parents independently and consistently reported a lack of signs of life after birth. Microscopically, the lung tissue did not show characteristic respiratory development or signs of physiological amniotic fluid aspiration during birth, but they were interspersed with bacterial cell nests and gas bubbles. Microbiological examinations revealed the gas-forming obligate anaerobic germ *F. gonidiaformans* in the lung tissue and in the heart blood. It is assumed that *F. gonidiaformans* was transmitted from the mother to the foetus, haematogenously or via the amniotic fluid. The foetus was stillborn with false-positive lung and gastrointestinal float test results.

## Key points

1. Fusobacterium gonidiaformans can produce (putrefactive) gases.
2. Intrauterine infection of the foetus by *F. gonidiaformans* with leucocytosis.
3. Putrefactive gases are produced prenatally.
4. ‘False-positive’ lung float sample due to putrefactive gases.

## Data Availability

The information in this publication is based on our own research and the literature cited in the bibliography.
